# Use of a Digital Medication Management System for Effective Assessment and Enhancement of Patient Adherence to Therapy (ReX): Feasibility Study

**DOI:** 10.2196/10128

**Published:** 2018-11-26

**Authors:** Ronit Shtrichman, Stefan Conrad, Kai Schimo, Ran Shachar, Ehud Machluf, Enrique Mindal, Howard Epstein, Shirli Epstein, Alan Paz

**Affiliations:** 1 DosentRx Ltd Har Tuv Israel

**Keywords:** medication adherence, medication nonadherence, personalized medicine, therapy management

## Abstract

**Background:**

Medication nonadherence is a major problem in health care, imposing poor clinical outcomes and a heavy financial burden on all stakeholders. Current methods of medication adherence assessment are severely limited: they are applied only periodically, do not relate to actual pill intake, and suffer from patient bias due to errors, misunderstanding, or intentional nonadherence. ReX is an innovative medication management system designed to address poor patient adherence and enhance patient engagement with their therapy. ReX controls and tracks pills from the point of packaging right through to the patient’s mouth. ReX generates robust, real-time adherence data. The system enables patients to report outcomes, complete surveys, and receive messages and instructions. ReX includes a reusable drug dispensing unit, disposable cassette containing pills, and a cloud-based data portal.

**Objective:**

We aimed to evaluate ReX feasibility by human factor studies including evaluation of ReX safety; ReX acceptance and usability; and ReX efficacy of providing pills according to a preprogrammed dose regimen, managing reminders and adherence data, and enhancing the adherence rate compared with the standard of care.

**Methods:**

The ReX system was evaluated in 2 human factor, nonclinical feasibility studies. Human subjects used ReX for the administration of pill-shaped Tic Tac sweets. The initial study evaluated ReX use and pill intake administration; second was a self-controlled, 4-day home-use study. All subjects took pills at home, according to a preprogrammed dose regimen, for 4 days each via the device (ReX test) or from standard packaging (control test). The adherence rate (percent of pills taken) was measured by the study subject’s report, remaining pills count, and ReX records (in the ReX test). ReX safety and usability were evaluated by a questionnaire filled out by the subject.

**Results:**

The initial feasibility study evaluated usability and acceptance of the ReX novel approach to pill dispensing. All subjects successfully managed 2 pill intakes. The ReX device was rated as easy to use by 81% (48/59) of subjects. The 4-day home-use study evaluated the safety, efficacy, and usability of the ReX system. No adverse event occurred; no pill overdose or pill malformation was reported. The overall adherence rate in the ReX test was 97.6% compared with 76.3% in the control test (*P*<.001). Real-time, personalized reminders provided in the event of a delay in pill intake contributed to 18.0% of doses taken during the ReX test. The ReX system was found easy to use by 87% (35/40) of subjects; 90% (36/40) felt comfortable using it for their medication.

**Conclusions:**

ReX’s novel “tracking to the mouth” technology was found usable and accepted by subjects. The assessment of adherence rates was reliable; adherence of subjects to the dose regimen was significantly enhanced when using ReX compared with the standard of care.

## Introduction

Medication nonadherence is defined as the extent to which patients fail to take medications or follow treatment recommendations as prescribed by their care providers. It is one of the most serious problems in health care, imposing a heavy financial burden on all stakeholders: insurers, employers, and patients [1].

The overall adherence for medication therapies was found to be almost 50% [2]. Forgetting to take medication and misunderstanding instructions are the most frequently reported reasons for nonadherence [3]. It is estimated that in the United States nonadherence leads to 125,000 deaths per annum and accounts for 33%-69% of all medication-related hospital admissions [4]. Between US $100 and US $300 billion of avoidable health care costs have been attributed to nonadherence in the United States annually, representing 3%-10% of total US health care costs [3]. A recent report estimated that nonadherence in 2016 cost the pharmaceutical industry up to $637 billion in lost sales, of which $250 billion were in the United States [5]. This estimate points to a far more significant problem than previously believed.

Adherence measurement is a considerable challenge. The current methods of measuring adherence may be classified as direct or indirect. Direct methods test the drug level or its metabolite in body fluids. Direct approaches are expensive, limited to periodic assessment, and subject to variations resulting from the patient’s condition at the time of test. Indirect methods include patient questionnaires, self-reports, pill counts, rates of prescription refills, assessment of patient’s clinical response, and patient diaries. Indirect methods are simple but inaccurate and biased [1].

Electronic medication packaging devices have been developed to remotely record, deliver, manage, and monitor drug intake information. The Medication Events Monitoring System can track and record the date and time of the medication removed from a container. The use of Medication Event Monitoring System was found to be reliable in several studies, at least compared with pill count and patients’ reports [6]. Other novel technological solutions involving cell phone apps aim to enhance adherence by providing alerts for pill intake according to the dose regimen. However, these technologies cannot track each pill or eliminate medication overdose and abuse [7].

ReX is an innovative medication management system designed to provide a comprehensive solution to the nonadherence problem. ReX monitors the drug from its packaging in the pharmacy through to its administration into the patient’s mouth. The pills are locked in the device and can be released only at the right time, at the specified dose, and only to the prescribed patient’s mouth. Pill intake data are recorded and transmitted in real time to caregivers. When a dose is missed, a personalized reminder is immediately provided to the patient. ReX can survey the patient’s well-being and be used as a treatment dairy. In this paper, we describe the evaluation of the ReX system in 2 human factor feasibility studies. The studies’ goals were to demonstrate its safety, efficacy, and usability in adherence assessment and enhancement.

## Methods

### ReX System Design

ReX is a hand-held, mobile device intended to provide solid oral medication on patient demand according to a preprogrammed treatment protocol. ReX aims to address poor patient adherence by providing personalized medication therapy management.

**Figure 1:**
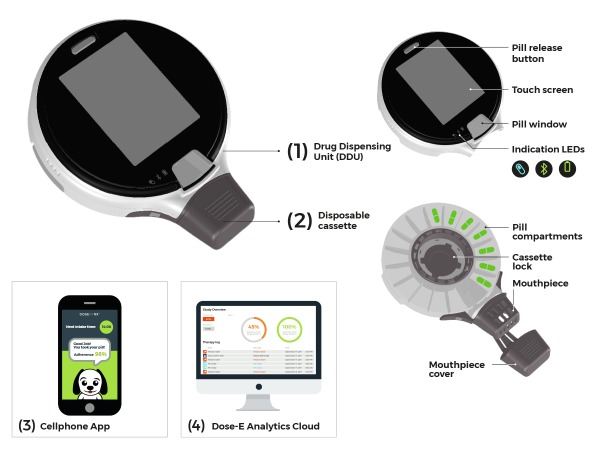
ReX system components.

The system comprises a reusable drug dispensing unit (DDU), a disposable cassette, a cellphone app, and a Dose-E Analytics cloud system. Figure 1 shows the ReX device, comprising reusable DDU (1), disposable cassette containing pills (2), cellphone app (3), and Dose-E Analytics cloud system (4). The DDU manages pill administration and includes a touch screen, which guides the user and presents patient-specific clinical surveys and therapy information. The DDU contains a chargeable battery and indicators demonstrating the device and the battery status, a pill window enabling pills to be viewed, operational sensors, and Bluetooth communication to an app on a cellphone. All therapy data are transferred to a patient-specific domain on a Web-based cloud. The DDU is also used to hold and lock the disposable cassette which contains the pills.

The disposable cassette is a locked, tamper-resistant container. It is supplied preloaded with bulk pills, located 1 in each of 16 separated pill compartments. The cassette is opened only on insertion in the DDU. The cassette includes an integral mouthpiece designed for pill ingestion. The mouthpiece incorporates an antichoke mechanism, which ensures that the pill falls directly onto the tongue. An integral protective cover keeps the mouthpiece is clean and sealed. Once empty, the cassette is automatically released by the device. Cassette exchange is easily performed by the user.

The cellphone app transfers data between the DDU and the Dose-E Analytics cloud. The Dose-E Analytics cloud system is a proprietary browser-based app in which all therapies and patient information are collected and managed. The cloud allows caregivers to set up and track the therapy online and follow the patient’s adherence. When a missed dose is recorded, the cloud sends alerts to a predefined contact person or to the call center.

### Principal Operation of ReX

#### Pill Intake Procedure

**Figure 2:**
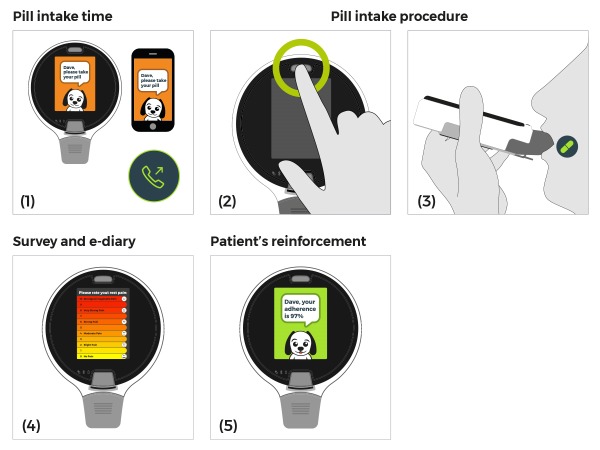
ReX operation and patient journey.

As seen in Figure 2, the DDU prompts the patient to take a pill at the defined time by means of sound, light, and animations and via the cellphone app (1). The patient requests a pill by pressing on the pill release button (2). The patient applies a slight suction on the mouthpiece and the pill is released onto his tongue (3). If the patient presses the button within the predefined lockout period, the device will not release a pill. If a delay is recognized, a personalized phone call reminder is provided. The device offers clinical surveys (4), recording of an e-dairy, therapy information, and reinforcements (5).

#### Data Management

The device records all pill intake events. This information is transmitted through the cellphone app to the Dose-E Analytics cloud. Therapy data can be relayed in real time to payers, providers, and caregivers.

#### Reminders and Alerts

The time window in which the user can take a pill is termed the tolerance time. The tolerance time determines the reminders, including visual and acoustic alerts, on the DDU screen and cellphone app. As the tolerance time window progresses without a pill being taken, the reminders escalate in frequency and intensity. Toward the end of the tolerance time, if a pill has still not been taken, an email is dispatched to the recognized contact person. The notified person contacts the patient by phone call to remind him to take his pill and to establish the cause of the delay. This process ensures that reminders are provided only when needed, eliminating diminished responsiveness to unsolicited alerts.

#### Surveys and Therapy Information

Real-time patient surveys and an e-dairy can be filled via the screen. The patient may use the screen to check his adherence rate, the course of treatment, and obtain treatment information (Figure 2).

### Initial Feasibility Study

#### Study Objectives

The initial feasibility study objectives were the evaluation of (1) ReX device functionality (inserting the cassette, pill extraction, screen menu) and (2) ease of extracting a pill and acceptance of the pill extraction concept. The study was nonclinical since the pills used were pill-shaped Tic Tac sweets.

#### Study Population

We enrolled 59 human subjects (29 males, 30 females), aged 18-92 years. The subjects were recruited following publication on social networks (LinkedIn, Facebook) and local advertisements. No compensation was provided to recruited subjects.

#### Study Design

All participated subjects were volunteers. All enrolled subjects signed an informed consent form. Each subject underwent a short one-on-one training session during which they were asked to insert a cassette and take 2 pills using the device. The subjects filled out a questionnaire about their experience with the ReX device.

#### Study Measures

The study evaluated the following parameters: subjects’ ability to insert a cassette, success rate of pill extraction using the device, understanding of screen menus, understanding the concept of lockout and overdose prevention, and overall ease of use. Results were recorded on a questionnaire comprising Likert-scale responses. Subjective and unsolicited opinions were noted.

### 4-day Home-Use Feasibility Study

#### Study Objectives

The objectives of the 4-day home-use feasibility study included

- Evaluation of the safety, efficacy, and usability of the ReX system in 4-day home use.
- Assessment of ReX ability to enhance adherence rate compared with standard of care (taking pills from standard pill container). Pill-shaped Tic Tac sweets were used to mimic medication. The study is, therefore, defined as nonclinical.

#### Study Population

We enrolled 40 human subjects, aged 18-90 years, and they all signed an informed consent form. The exclusion criteria were significant physical disability or mental disorder and failure to extract 2 pills after 3 attempts during ReX training. Subjects were recruited following publication on social networks (LinkedIn, Facebook) and local advertisements. No compensation was provided to the recruited subjects.

#### Study Design

In this self-controlled study each subject participated in the following sequential tests:

Control test: Subjects took pills from the original package and manually reported for each pill intake or missed dose. No reminders were performed during this test. At study end, the remaining pills were counted.

**Figure 3:**
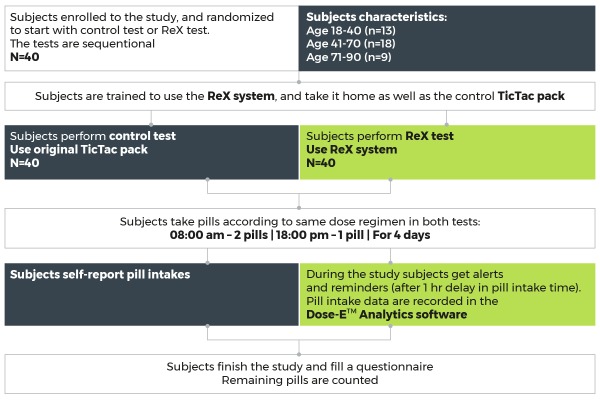
Flow diagram of ReX 4-day home-use feasibility study.

ReX test: Subjects took pills using the ReX device. Delays in pill intake lead to real-time personalized reminders. At study end, the remaining pills were counted and compared with the ReX records. Subjects were asked to report any safety or functionality problem encountered during the study and to fill out a questionnaire regarding their experience with ReX (Multimedia Appendix 1). The study design is shown in Figure 3.

Both tests had the same duration and dose regimen of 2 pills in the morning and 1 pill in the evening, for 4 days. The specific time of pill intake was programmed in the ReX device as 08:00 am and 18:00 pm. The tolerance time was set as ±1 hour. In case of pill intake delay after the tolerance time, an email was dispatched prompting the principal investigator to contact the subject and remind him to take the missing pill.

Before the study start, each subject underwent a short, in-person training session in which he successfully completed 2 pill intakes using the ReX. During the ReX test, real-time adherence data were communicated to the Dose-E Analytics cloud and made available to the study’s principal investigator.

### Statistical Analysis

The adherence rate was calculated as percent of doses taken. In the ReX test, percent doses taken before and after the reminder were calculated and included in the adherence rate. Paired differences were calculated for adherence rate and percent of missed doses between the ReX test and control test for all subjects and by age categories. The paired *t* test and nonparametric signed-rank test for 2 means (paired observations) were applied to analyze the paired differences. All tests were 2-tailed, and a *P* value ≤5% was considered statistically significant. The data were analyzed using SAS 9.3 (SAS Institute, Cary North Carolina).

## Results

### ReX Initial Feasibility Study

The initial feasibility study aimed to evaluate usability, acceptability, and ease of use of the ReX device for oral medication provision. There were 59 subjects, aged 18-92 years, in the study (Table 1).

Following a short tutorial, all subjects successfully inserted the cassette into the DDU and defined the process as easy. The usability of ReX for pill extraction was measured by the success rate of 2 pill intakes. All subjects managed 2 successful attempts at pill intake as required, and 81% (48/59) of subjects required only 1-2 attempts to extract a pill. A learning effect was evident in taking the pills: subjects were more successful in taking their second pill compared with the first.

All subjects easily grasped the concept and functionality of the screen displays. After 2 successful attempts at pill intake, 100% (59/59) of subjects understood the concept of lockout and overdose prevention, as confirmed by a third attempt at pill intake. The overall impression was very positive, with 97% (57/59) of subjects expressing confidence in using ReX by themselves and without assistance.

**Figure 4:**
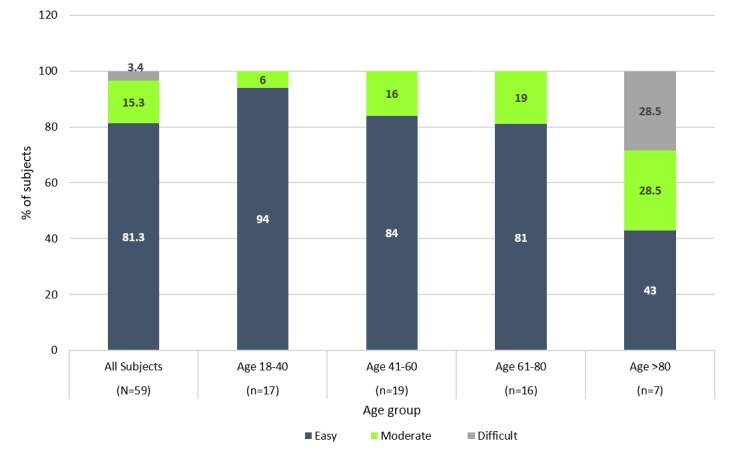
Usability of ReX device.

Figure 4 demonstrates subjects’ response regarding overall ReX ease of use: 81% (48/59) of all subjects rated the ReX device as easy to use. This rating did not appear to be influenced by years of formal education, as 100% of subjects with 6-10 and >20 years of formal education defined the ReX use as easy, while 4%-8% of subjects with 11-15 and 16-20 years of formal education, respectively, defined it as difficult.

However, analysis by age group demonstrated that ReX usability is influenced by age: 29% (2/7) of subjects >80 years old reported that ReX was difficult to use. Opinions as to ease of use slightly decreased with age. Still, 94% (16/17) of subjects aged 18-40 and 81% (42/52) of subjects aged up to 80 years defined the ReX as easy to use.

### 4-day Home-Use Feasibility Study

This study aimed to evaluate ReX’s usability during home use and its capability to monitor and enhance patient adherence. The study was designed as self-controlled: pill intake using ReX was compared with intake from a standard pill container as the control. The same dose regimen was used for both methods. We enrolled 40 subjects with an age range of 18-90 years, as described at Table 2.

**Table 1:** Demographic characteristics of 59 human subjects participating in the initial feasibility study.

Group		Ages (years), n (%)							
		All subjects	18-30 (n=10)	31-40 (n=7)	41-50 (n=11)	51-60 (n=8)	61-70 (n=10)	71-80 (n=6)	>81 (n=7)
**Gender**									
	Male	29 (49)	4 (7)	6 (10)	7 (12)	3 (5)	4 (7)	2 (3)	3 (5)
	Female	30 (51)	6 (10)	1 (2)	4 (7)	5 (8)	6 (10)	4 (7)	4 (7)
**Years of formal education**									
	6-10	4 (7)	1 (2)	3 (5)	—^a^	—	—	—	—
	11-15	24 (41)	7 (12)	—	3 (5)	2 (3)	4 (7)	3 (5)	5 (8)
	16-20	26 (44)	1 (2)	3 (5)	7 (12)	4 (7)	6 (10)	3 (5)	2 (3)
	>20	5 (8)	1 (2)	1 (2)	1 (2)	2 (3)	—	—	—
Take pills regularly		29 (49)	3 (5)	2 (3)	5 (8)	3 (5)	6 (10)	5 (8)	5 (8)

^a^Not applicable.

**Table 2:** Demographic characteristics of 40 human subjects participating in the 4-day home-use study.

Group		Ages (years), n (%)						
		All subjects	18-40 (n=13)	41-70 (n=18)			71-90 (n=9)	
Age (years), mean (SD)		48.7 (20.2)	27.5 (6.6)		53.7 (8.5)			79.4 (5.3)
**Gender**								
	Male	21 (53)	6 (15)	10 (25)			5 (13)	
	Female	19 (48)	7 (18)	8 (20)			4 (10)	
Take pills regularly		17 (43)	1 (8)		10 (56)			9 (100)

**Table 3:** Adherence rate statistical analysis for all users and by age group.

Adherence Rate		N	Mean (SD)	Min	Median	Max	Lower 95% CI	Upper 95% CI	*P* value (paired *t* test)	*P* value (signed-rank test)
**All subjects**									<.001	<.001
	ReX	40	97.6 (5.2)	83.3	100.0	100.0	95.9	99.3	—^a^	—
	Control	40	76.3 (24.6)	0.0	83.2	100.0	68.6	84.0	—	—
**Age, 18-40 years**									.002	.004
	ReX	13	98.1 (4.7)	87.5	100.0	100.0	95.2	100.9	—	—
	Control	13	64.9 (27.8)	0.0	66.7	100.0	48.9	80.9	—	—
**Age, 41-70 years**									.02	.008
	ReX	18	96.8 (5.4)	87.5	100.0	100.0	93.9	100.4	—	—
	Control	18	79.4 (25.7)	8.3	85.4	100.0	65.7	93.1	—	—
**Age, 71-90 years**									.02	.03
	ReX -C	9	98.6 (5.8)	83.3	100.0	100.0	93.9	101.2	—	—
	Control	9	86.2 (13.4)	66.7	87.9	100.0	77.0	94.0	—	—

^a^Not applicable.

#### ReX Device Safety

The safety of the ReX system was evaluated by a questionnaire filled out by the subjects and confirmed by data recorded in the Dose-E cloud. No incidence of pill overdose dispensed occurred, and no pill malformation was reported. Furthermore, no severe adverse events, such as pill inhalation, occurred.

#### ReX Device Efficacy

The functionality of the ReX system was measured by the success rate of pill intakes. All subjects (40/40, 100%) successfully obtained pills by the ReX device according to their dose regimen. The principal investigators and 80% of subjects (32/40) did not encounter any technical difficulties during device use, such as problems involving the touch screen; pills extraction on time; and data transfer, monitoring, and management by the Dose-E Analytics cloud system.

The 2 processes of pill administration were compared: use of the ReX system (ReX test) or use of a standard pill container (control test). The subject’s adherence rate was measured by the subject’s report, remaining pill count, and ReX record (only for ReX test).

Table 3 lists the mean adherence rate obtained for all subjects and for the 3 different age groups. Results show that the adherence rate of all subjects in the control test was 76.3% while the adherence rate in the ReX test was 97.6% (*P*<.001). Analysis by age group also demonstrated significantly higher adherence rates in the ReX test compared with the control test. The adherence rate in ReX test was stable and reached 97%-98% for all age groups with very low variations (up to 5.2%). In contrast, adherence rates in the control test varied significantly between age groups and were subject to high SDs (up to 24.6%). Adherence rates in the control tests were 64.9%, 79.4%, and 86.2% for age groups of 18-40 (*P*<.001), 41-70 (*P*=.02), and 71-90 (*P*=.02) years, respectively.

Following a 1-hour delay in pill intake recorded by the ReX system (1-hour delay was defined as beyond the tolerance time), subjects doing the ReX test received a personalized reminder (phone call) from the principal investigator. This personalized communication aimed to prompt them to take their delayed dose and to understand the cause of the delay. It was found that 18% of doses were taken after personalized reminders. Only 2.4% of doses were completely missed in the ReX test, while 23.7% of doses were missed in the control test.

**Figure 5:**
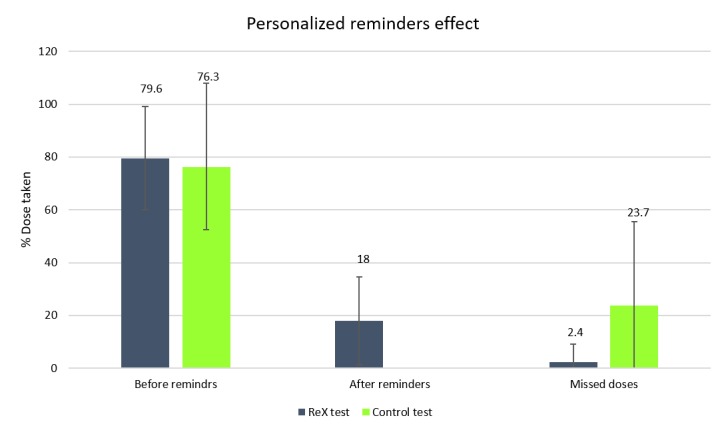
Effect of real-time, personalized reminders on percent dose taken.

Doses taken were recorded by pill count, self-report at study end, and by ReX record (only in ReX test). Figure 5 shows percent of dose taken before personalized reminders in both tests, after personalized reminders only in the ReX test (personalized reminders are not applicable in the control test), and percent of missed doses in both tests.

### ReX Device Usability

The usability and ease of use of the ReX system were evaluated by questionnaires completed by the subjects (Multimedia Appendix 1), of whom 87% (35/40) found the ReX system easy to use, and 90% (36/40) mentioned that they felt comfortable using ReX for their medications. Moreover, when comparing between the ReX device and standard package, subjects responded that the ReX device was more effective in reminder provision (36/40, 90%) and in error prevention (38/40, 95%), and the ReX device was preferred to keep an e-dairy during medication therapy (33/40, 82%).

## Discussion

### Principal Findings

ReX is an innovative system designed to manage oral medication therapy by directly monitoring pill intakes, allowing high confidence in the resulted adherence rate. ReX incorporates a “tracking to the mouth” approach. This is based on a patented technology for the safe ingestion of solid pills into the patient’s mouth and digitally tracking this action to provide accurate, reliable, and real-time adherence data to stakeholders. Electronic monitoring devices have been shown to provide good-quality information on adherence rate [8] and found to hold promise of improving adherence [9-11]. Methods that involve reinforcement interventions have been successful in improving patients’ cooperation and adherence behaviors. Clear and effective communication between caregivers and their patients has been found to be essential in improving patients’ adherence [12].

An initial feasibility study was conducted to evaluate the basic usability parameters of the ReX device and acceptability of the pill extraction concept. Results demonstrated that all subjects could successfully use the device for pill intake. The device was defined as easy to use, and 81% (48/59) of subjects required only 1-2 attempts for successful pill intake. Only mature users (aged >80 years) reported more difficulty, although they all could manage and extract pills using the device. These results demonstrate the feasibility of the ReX novel technology.

Following this, we designed a 4-day home-use study to evaluate ReX safety, efficacy, and usability. The adherence rate by ReX was compared with the standard of care. The adherence rate was tested by subjects’ reports, remaining pill count, and by ReX records (during the ReX test). Although patient self-report and remaining pills counts are common methods to assess patient adherence, there is extensive evidence that such methods greatly overestimate medication adherence when compared with plasma drug levels and electronic device measurements [8,13,14]. These methods may also suffer from intentional nonadherence, including removing and discarding pills from a blister card or bottle, to create false records while reporting good adherence [8]. In contrast, the ReX approach eliminates false measurements since each pill is tracked directly during ingestion. The adherence rate is obtained in an unbiased way, without patient involvement.

The 4-day home-use feasibility study demonstrated that ReX device is safe: no adverse events, overdoses, or pill malformations were encountered. The safety of pill ingestion by sucking was previously confirmed in a clinical study evaluating the same technology for pain analgesic medication provision to postoperative patients in the hospital setting [15].

Functionality analysis revealed that all subjects could successfully use the ReX device for pill intake and that adherence data were available for the study’s principal investigators in real time. Study results showed a statistically significant difference of 21.3% in adherence rate between the ReX test and the control test (97.6% and 76.3%, respectively). It is possible that low adherence rates in the control test occurred because subjects took Tic Tac sweets and not real medication, making it less important to them. However, the same subject group achieved 97.6% adherence rate in the ReX test. Such high adherence was due to stringent monitoring of each dose by the study’s principal investigator and timely reminders to subjects in any case of delayed dose. This created effective communication and reinforcement to take the missed dose.

The adherence rate of the control test varied between the 3 different age groups of 18-40, 41-70, and 71-90 years. Only 8% (1/13) of the young subjects (age 18-40 years) took pills regularly and were, therefore, not used to taking pills. Their adherence in the control test was consequently relatively low (64.9%). However, use of ReX increased their adherence rate to 98.1%. Mature subjects (age 71-90 years) demonstrated higher adherence in the control test (86.2%). This may be because all subjects (9/9, 100%) of this age group take pills on a daily basis. However, using ReX enhanced adherence rate in all age groups. All differences in adherence rate between the ReX test and control test were statistically significant.

The ReX system also demonstrates benefits over technological solutions of adherence assessment and enhancement. An available approach is a memory chip embedded in bottle caps or blister packs that tracks medication adherence electronically. For example, the Medication Event Monitoring System cap [9] (AARADEX Group, SA), which records the date and time of each opening. However, since this system does not track the intake of each pill, a false record of dosing can easily be created [8]. A vast pool of medication adherence cellphone apps is also available to help patients manage their medication regimen [16]. However, these apps add a burden on subjects to record and update each time they take a pill. This action may be missed at the real time of pill intake. Also, usual app alerts may be ignored and missed by subjects while in routine use.

During the 4-day home-use ReX study, personalized reminders were shown to add 18% of doses taken. This explains the major difference in adherence rate between the ReX test and the control test. Notably, adherence rates were almost similar between these tests before any personalized reminder. This highlights the effect of personal reminders provided in real time and only when needed. It also confirms the minimal impact of conventional visual and acoustics alerts that automatically appeared and are often ignored by the user.

The final percent of missed doses in the ReX test (2.4%) was almost 10-fold lower than in the control test (23.7%). This observation clearly demonstrates the benefit of using ReX system to monitor and enhance adherence.

The usability of ReX was evaluated by questionnaires filled out by the subjects participating in both the ReX and control tests. After 4 days of use and 12 pill intakes, 90% (36/40) of subjects reported that they felt comfortable taking their medication via ReX, and 87% (35/40) of subjects mentioned that it was easy to use. Moreover, most subjects believed that ReX provided effective reminders (90%), was highly effective in error prevention (94%), and was most suitable to be used as an e-dairy to record symptoms during therapy (82%). These results are in agreement with the high usability and acceptance of the technology as demonstrated in a previous clinical study [15].

The feasibility studies described here demonstrate the potential of the ReX system for medication management. ReX may provide a considerable benefit in medication therapies such as: high risk drugs, to eliminate errors, overdose, and abuse (eg, opioid treatment [17], anticoagulants, or stimulants for attention-deficit/hyperactivity disorder treatment [18]); high cost drugs (eg, specialty drugs [19]); and clinical trials, in which adherence critically affects outcome reliability and study cost [20].

In summary, ReX is an innovative solution providing reliable, unbiased, and cost-effective adherence monitoring and enhancement, while safeguarding the patient by elimination of medication errors, overdose, and abuse.

### Conclusions

Two feasibility studies confirmed the safety, efficacy, and usability of the ReX system. All objectives were achieved. Regarding ReX safety, the ReX system was safe under the study conditions; no adverse events, no pill provision during the lockout interval, no overdose, and no pill malformation were found. Evaluation of ReX efficacy demonstrated that all subjects successfully used ReX to take the pills according to their dose regimen. The data were available to the study’s principal investigator in real time, and personalized reminders were provided in any case of a 1-hour delay in pill intake. The adherence rate in the ReX test was 97.6%, significantly higher compared with the control test (76.3%). The effectiveness of real-time personalized reminders was indicated by 18% of doses in the ReX test being taken after the reminders were received by the study subjects. As for ReX usability, ReX technology was well accepted by subjects participating in the studies. Over 80% of subjects described it as easy to use and mentioned that they felt comfortable to use it for their medications.

### Study Limitations

The limitations of the study included the heterogeneous small group sizes and the use of candies and not real drugs. Also, Tic Tac sweets are chewable and are not swallowed with water like standard drugs. The adherence rate was based on self-reporting and remaining pill counts in the control test. These are known to be unreliable methods. ReX records are more reliable in the ReX test. The study design ensured that half of the subjects completed the control test before the ReX test and vice versa for the other half.

## Appendix

Multimedia Appendix 1 ReX Use Questionnaire.

## Abbreviations

DDU: drug dispensing unit
