# Supplementary material for: Use of a Digital Medication Management System for Effective Assessment and Enhancement of Patient Adherence to Therapy (ReX): Feasibility Study
Source: JMIR Hum Factors. 2018 Nov 26;5(4):e10128. doi: 10.2196/10128 (PMC6288592; doi:10.2196/10128)
Supplement: Multimedia Appendix 1 [file humanfactors_v5i4e10128_app1.pdf]

Multimedia Appendix 1

ReX Use Questionnaire:

% of subjects selecting the specific answer is shown in brackets.

Training:

1. Was the on-screen training tutorial, including cassette exchange, pill intake instructions, device recharging and connectivity, clear?
   Very clear (66%)
   Clear (31%)
   Not so clear (3%)
   Very unclear (0)

2. Following the on-screen tutorial, were you able to use the ReX device without the trainer's help?
   Yes (81%)
   Not sure (12%)
   No (7%)

Reminders:

3. If you missed a pill, the reminder escalation concept was activated. Did you find the following reminders useful? (multiple choices)

- *Visual reminder – device blinking (8%)*
- *Acoustic reminder – device beeping (54%)*
- *Cell phone text messages (34%)*
- *Phone call from the trainer (48%)*
- *I didn't need reminders (18%)*

4. Which reminders irritated you? (multiple choices)

- *Visual reminder – device blinking (0%)*
- *Acoustic reminder – device beeping (5%)*
- *Cell phone text messages (0%)*
- *Phone call from the trainer (8%)*
- *None of the above (90%)*

Safety:

5. Did you encounter any of the following safety problems during the study?

- Pills overdose – more than one pill dispensed at a time?
  Yes (0%)
  No (100%)

- Pill falling down during sucking and therefore did not reach your mouth.
  Yes (2%)
  No (98%)
- Pill malformation or broken.
  Yes (0%)
  No (100%)

Functionality:

6. Did you encounter any technical problems with the ReX device?
   Yes (19%)
   No (81%)

Usability:

7. Please compare the two methods you used for pill intake, ReX device or current practice (standard package)?

- Which method was more effective in reminding you to take your dose?
  ReX device (89%)
  Current practice (I don't need reminders) (11%)
- Which method was more effective in preventing errors?
  ReX device (94%)
  Current practice (6%)
- If you had to document medication intake and side effects in a diary during treatment, which method would you prefer?
  ReX device (82%)
  Current practice (documenting long hand) (18%)

8. When carrying the ReX with you, was the ReX a comfortable size/weight?
   Yes (57%)
   No (43%)
9. Having completed the study, would you feel comfortable using ReX?
   Yes (89%)
   No (11%)
10. How do you rate overall ease of use?
    Easy (87%)
    Moderate (13%)
    Difficult (0%)
